# Natural Recovery by the Liver and Other Organs after Chronic Alcohol Use

**DOI:** 10.35946/arcr.v41.1.05

**Published:** 2021-04-08

**Authors:** Paul G. Thomes, Karuna Rasineni, Viswanathan Saraswathi, Kusum K. Kharbanda, Dahn L. Clemens, Sarah A. Sweeney, Jacy L. Kubik, Terrence M. Donohue, Carol A. Casey

**Affiliations:** 1 Department of Internal Medicine, Section of Gastroenterology, University of Nebraska Medical Center, Omaha, Nebraska; 2 Research Service, U.S. Department of Veterans Affairs Nebraska-Western Iowa Health Care System, Omaha, Nebraska; 3 Department of Internal Medicine, Section of Diabetes, Endocrinology, and Metabolism, University of Nebraska Medical Center, Omaha, Nebraska; 4 Department of Internal Medicine, Section of Cardiovascular Medicine, University of Nebraska Medical Center, Omaha, Nebraska; 5 Fred & Pamela Buffet Cancer Center, University of Nebraska Medical Center, Omaha, Nebraska

**Keywords:** alcohol use disorder, alcohol-associated liver disease, alcohol abstinence, alcohol cessation, alcohol, alcoholic pancreatitis, alcoholic cardiomyopathy

## Abstract

Chronic, heavy alcohol consumption disrupts normal organ function and causes structural damage in virtually every tissue of the body. Current diagnostic terminology states that a person who drinks alcohol excessively has alcohol use disorder. The liver is especially susceptible to alcohol-induced damage. This review summarizes and describes the effects of chronic alcohol use not only on the liver, but also on other selected organs and systems affected by continual heavy drinking—including the gastrointestinal tract, pancreas, heart, and bone. Most significantly, the recovery process after cessation of alcohol consumption (abstinence) is explored. Depending on the organ and whether there is relapse, functional recovery is possible. Even after years of heavy alcohol use, the liver has a remarkable regenerative capacity and, following alcohol removal, can recover a significant portion of its original mass and function. Other organs show recovery after abstinence as well. Data on studies of both heavy alcohol use among humans and animal models of chronic ethanol feeding are discussed. This review describes how (or whether) each organ/tissue metabolizes ethanol, as metabolism influences the organ’s degree of injury. Damage sustained by the organ/tissue is reviewed, and evidence for recovery during abstinence is presented.

## INTRODUCTION

A vast body of evidence from human studies and animal research clearly indicates that chronic, heavy alcohol consumption causes structural damage and/or disrupts normal organ function in virtually every tissue of the body. In heavy consumers of alcohol, the liver is especially susceptible to alcohol-induced injury.^1^,^2^ Additionally, several other organs—including the gastrointestinal (GI) tract, pancreas, heart, and bone—exhibit impaired function after chronic ethanol use.^3^

As the largest internal organ and the first to see blood-borne nutrients, toxins, and xenobiotics absorbed from the GI tract, the liver is especially vulnerable to alcohol-induced damage. The liver plays a key role in the body’s metabolic regulation and is a “frontline” organ that rapidly metabolizes (i.e., chemically converts or oxidizes) alcohol to less harmful substances. However, acetaldehyde, the first metabolite generated by alcohol oxidation is actually more toxic than alcohol, but acetaldehyde is rapidly converted to acetate for use in other biochemical reactions in the body.^3^ Thus, although the liver has the capacity to eliminate toxic substances, continual excessive alcohol consumption can seriously damage the liver and other organs. Recent studies report that alcohol-associated liver disease (ALD) is one of the leading preventable causes of illness and death from liver disease in the United States and the world.^4^

After drinking stops, damaged organs may regain partial function or even heal completely, depending on the extent of organ damage and whether there is relapse (i.e., resumption of drinking). Organ damage due to heavy drinking is greatest in the liver, in part because the liver has higher levels of enzymes that catalyze the metabolism of acetaldehyde from alcohol. Acetaldehyde is more toxic than ethanol because it is highly reactive and binds to biomolecules (e.g., proteins, lipids, nucleic acids) and disrupts their function.^3^,^5^ However, even after years of chronic alcohol use, the liver has remarkable regenerative capacity and, after sustained cessation of drinking, can recover a significant amount of its original mass.^6^

This review examines injury to selected organs and tissues from chronic alcohol use and their “natural recovery” after drinking ceases. Data have been obtained from both human studies and studies with experimental animal models of alcohol administration. The main points of emphasis will be how ethanol, the active ingredient and principal component in alcoholic beverages, affects the liver, GI tract, pancreas, heart, and bone. This review describes how (or whether) each organ/tissue metabolizes ethanol, as this property is closely related to the organ’s degree of injury. The damage sustained by the organ/tissue is then described, and the evidence for natural recovery after drinking cessation is reviewed. It is important to emphasize that “natural recovery” is that which is unaided by external agents that directly enhance healing of the damaged organ or tissue. In the case of the liver, such agents include drugs or other compounds that suppress inflammation or dietary or medicinal compounds (e.g., betaine, caffeine, aspirin), which alleviate tissue damage by enhancing protective pathways, thereby preventing further damage. Throughout the article, “alcohol” and “ethanol” are used interchangeably, given that they have the same meaning.

## LIVER

### Alcohol Metabolism in the Liver

In humans (and other animals, such as rodents), the liver is the primary site of alcohol metabolism. The same two enzymes catalyze ethanol oxidation in both species. The major, most catalytically efficient enzyme is alcohol dehydrogenase (ADH), which catalyzes the formation of acetaldehyde from alcohol. The other enzyme, cytochrome P450 2E1 (CYP2E1), is catalytically less efficient than ADH, but it increases in both content and activity severalfold after continual alcohol exposure.^3^ This increase, called “induction,” further accelerates alcohol conversion to acetaldehyde, which is rapidly detoxified by its conversion to acetate by the enzyme aldehyde dehydrogenase (ALDH).^3^,^7^ Many laboratories utilize rodent models to examine ALD to elucidate the mechanisms responsible for such injury. As in humans, fatty liver (steatosis) is the earliest pathophysiological change that occurs in rodent livers after chronic alcohol administration. In rodent models, with continued drinking, hepatic steatosis can worsen to further injury such as alcoholic steatohepatitis (ASH). Fibrosis and cirrhosis occur when nutrients such as choline and/or methionine are deleted from the diet, when an endotoxin is simultaneously administered to increase injury, or after continual intragastric infusion of high levels of alcohol in liquid diets.^8^ Other studies have administered alcohol to nonhuman primates (baboons) to induce liver fibrosis.^9^ However, most laboratories utilize rodent models, which are more manageable and can be used in greater numbers than nonhuman primates.

### Liver Injury and Recovery After Chronic Alcohol Use in Humans

Fatty liver (steatosis), characterized by an accumulation of lipids in hepatocytes, is one of the earliest pathological changes in the progression of ALD. More than 90% of people who drink heavily consume up to 60 grams or more of ethyl alcohol per day. Most of these individuals develop fatty liver.^2^ Once the liver becomes steatotic, it is more prone to damage by inflammatory mediators (tumor necrosis factor, endotoxin) and/or toxic agents, leading to progression to ASH, fibrosis, and eventually cirrhosis and, in some cases, hepatocellular carcinoma. Even though virtually all heavy-alcohol consumers develop fatty liver, only about 20% to 40% of such people develop steatohepatitis, and a subset of these latter individuals develop the more advanced stages of ASH, cirrhosis, and hepatocellular carcinoma.^10^ Progression to further injury depends on the genetic constitution of individuals, their lifestyle (diet and exercise), and their exposure to viral infections, all of which contribute to disease progression and severity.^11^ The actual mechanisms involved in ALD development are complex and multifactorial, including gut and other tissue dysfunctions that influence liver pathology. Other parts of this review describe such dysfunctions in greater detail. Abstinence from alcohol is considered the most effective therapeutic strategy to recover from ALD, and there is clear evidence that abstinence can improve outcomes at nearly all stages of this disease.^6^

#### Diagnosis and recovery from ALD steatosis

Excessive use of alcohol (≥ 60g/day) for more than 2 weeks results in development of fatty liver (steatosis), characterized by deposition of fat in more than 5% of hepatocytes resulting in mostly macrovesicular steatosis (large intrahepatocyte lipid droplets) with or without minimal inflammation. Steatosis is mostly asymptomatic, although some people feel weakness, nausea, and pain in the right upper quadrant. Mild elevations in serum alanine transaminase (ALT), aspartate transaminase (AST), and gamma glutamyl transferase (GGT) are seen in patients with ALD. After abstinence from alcohol for 2 to 3 weeks, hepatic steatosis completely resolves and liver biopsies appear normal when examined by electron microscopy.^12^ Similarly, Mehta et al. reported that 1 month of abstinence from alcohol by heavy-alcohol consumers (average consumption ~258 g/week) reduced serum ALT, AST, GGT, and carbohydrate-deficient transferrin to baseline (abstinence) levels.^13^ In addition, insulin resistance, systolic and diastolic blood pressure, and serum cholesterol levels were also reduced with abstinence from alcohol. These changes were attained without significant lifestyle adjustments such as changes in diet or increased exercise, indicating that abstinence was the major factor in recovery.^14^

#### Alcoholic steatohepatitis

With continued excessive drinking, about 20% to 40% of heavy-alcohol consumers with steatosis develop alcoholic steatohepatitis (ASH), characterized by fatty liver, inflammation with accumulation of neutrophils, ballooning degeneration of hepatocytes with or without Mallory-Denk bodies, and pericentral and perisinusoidal fibrosis. The severity of ASH can range from mild to severe and is superimposed on chronic liver disease. Severity of ASH can be assessed by the model for end-stage liver disease (MELD). A MELD score greater than 20 has been proposed as defining severe ASH with approximately 20% mortality.^1^ Steatohepatitis symptoms include reduced appetite, nausea and vomiting, abdominal pain, fatigue, and weakness. People with severe alcoholic hepatitis exhibit jaundice (yellowing of the skin), dark urine, kidney failure, and confusion. ASH is diagnosed by a serum AST:ALT ratio greater than 1.5:1 with absolute ALT and AST numbers not exceeding 400 international units per liter, increased GGT, serum bilirubin greater than 3 mg/dl, and documented heavy alcohol use until 8 weeks prior to seeking help.^15^ Ultrasound and magnetic resonance analyses are additionally used to confirm ASH. Currently, hepatologists recommend liver biopsies for diagnosis of ASH, as one-third of patients who are asymptomatic can show advanced fibrosis histologically.^10^ As for steatosis, the major therapy recommended for mild ASH and severe ASH with systemic inflammatory response syndrome is abstinence from alcohol consumption. This provides the best long-term outcome for survival and recovery. Indeed, Kirpich et al. (2017) reported that after 2 weeks of abstinence, patients who presented with inflammation and increased serum endotoxin showed improvement, as indicated by decreased serum AST, ALT, and cytokeratin 18 (a sensitive marker of liver injury), as well as lower levels of tumor necrosis factor alpha and endotoxin.^16^ In other articles in this topic series, information is given on pharmacological therapy, in addition to cognitive behavioral therapy, which is known to be key to preventing relapses during abstinence; both of these therapies show increased recovery from ALD.^6^ In addition, nutritional supplementation is beneficial for recovery from ALD.^10^

#### Fibrosis and cirrhosis

Repeated episodes of ASH are accompanied by hepatic fibrosis and characterized by ballooned and dying hepatocytes and abnormal deposition of extracellular matrix around these cells. The stage/intensity of fibrosis (F0–F4) can be evaluated histologically and, in some cases, on the basis of liver stiffness, which is determined by transient elastography (FibroScan).^17^ When overexposed to alcohol, the liver loses its efficiency, and inflammatory damage produces scar tissue and fatty deposits in the organ. Normal liver parenchymal cells are replaced by regenerative nodules surrounded by fibrotic (scar) tissue. If enough scar tissue develops, the liver loses function in those scarred areas. Decompensated liver cirrhosis occurs when the liver can no longer properly perform its functions because of excessive scar tissue. Symptoms include fatigue, spider angioma (radiating blood vessels beneath the skin), palmar erythema (reddening of the palms), and jaundice (yellowing of the skin). These patients also have an increased risk of developing hepatocellular carcinoma, with a lifetime risk of about 3% to 10% and an annual risk of about 1%. The American College of Gastroenterology recommends that patients with alcohol-associated cirrhosis undergo screening with ultrasound examination every 6 months.^18^ At this stage, abstinence from alcohol improves survival rates.^6^,^14^,^19^

### Liver Injury and Recovery After Alcohol Administration in Animals

Researchers have studied molecular mechanisms of ALD and recovery from ALD in several animal models, most notably in rats and mice, using a wide variety of experimental conditions and various genetic backgrounds. As noted previously, both rats and mice develop fatty liver after alcohol administration, but progression to fibrosis or cirrhosis occurs only with manipulation of the diet and/or injection of an agent such as endotoxin or low-dose carbon tetrachloride to enhance a fibrotic response. This review summarizes cellular mechanisms that contribute to resolution of liver injury in alcohol-fed rats subjected to alcohol cessation. All studies described here used a similar model to investigate effects of alcohol and its cessation: Rats fed control or alcohol-containing Lieber-DeCarli liquid diets for 1 to 6 weeks showed typical serum alcohol concentrations of 200 to 300 mg/dl.^20^–^22^ Subsequently, randomly chosen alcohol-fed rats were weaned from the alcohol diet.^21^–^23^

#### Receptor-mediated endocytosis

Work from Casey and others has identified alcohol-induced defects in protein trafficking and organelle function, both of which recover upon alcohol cessation.^21^,^24^ The latter studies focused on the asialoglycoprotein receptor, a hepatocyte-specific receptor, which exhibits decreased function after even 1 week of alcohol administration.^21^ The authors identified impaired binding, internalization, and degradation of several ligands internalized by receptor-mediated endocytosis. In all cases, recovery to control levels of receptor-mediated endocytosis by the asialoglycoprotein receptor was partially restored after 2 to 3 days of refeeding with the control diet, and function was fully restored after 7 days of refeeding. These findings suggest that the detrimental effects of alcohol on protein trafficking pathways occur rather rapidly (1 to 5 weeks) and that complete recovery is obtained within 7 days after cessation of alcohol consumption.

#### Golgi apparatus organization

Another study reported that alcohol cessation normalizes alcohol-induced Golgi apparatus disorganization in the liver.^25^ These findings further support the notion that alcohol cessation reverses alcohol-induced trafficking defects. Here, chronic alcohol administration caused de-dimerization of the large Golgi matrix protein giantin in rat hepatocytes, leading to Golgi apparatus disassembly. Alcohol cessation and refeeding with the control diet for 10 days restored the compact, native structure of the Golgi apparatus.

#### Mg^2+^ levels

In another study, Torres et al. reported that 3 weeks of alcohol administration to rats impairs hepatocytes’ ability to increase the level of magnesium ion (Mg^2+^) in the extracellular compartment. Ten days after alcohol cessation, Mg^2+^ homeostasis was restored.^23^

#### Steatosis

Additionally, resolution of alcohol-induced fatty liver after alcohol cessation has been reported. Here, alcohol feeding increases hepatic triglycerides, confirmed by microscopic analyses of liver sections, which clearly show lipid droplet accumulation associated with elevated levels of ADH, CYP2E1, and lipid peroxides, as well as higher levels of serum AST, ALT. and nonesterified fatty acids (NEFA, or free fatty acids).^22^ After alcohol removal and refeeding with the control diet, there was normalization of serum NEFA and ALT levels with a significant (but not complete) reduction of hepatic triglycerides. The latter reduction was associated with normalization of ADH and CYP2E1 to control levels. Additionally, there was concomitant reduction of hepatic lipid peroxides, indicating lower levels of oxidants.^22^ These findings reveal that alcohol cessation attenuates generation of oxidants to alleviate hepatocyte damage, as confirmed by normalization of ALT levels.

#### NEFA levels

It is well established that impaired liver function affects other organs, and vice versa. For example, high serum NEFA levels in alcohol-fed rats arise from alcohol-induced lipolysis in adipose tissue, generating serum NEFA levels that exacerbate hepatic fat accumulation. This occurs because hepatocytes rapidly take up circulating NEFA,^22^ which, upon their entry into hepatocytes, are esterified with glycerol to form triglycerides. Notably, alcohol removal and refeeding with the control diet normalize serum NEFA levels, indicating that alcohol cessation slows the hepatic uptake of circulating fatty acids and attenuates adipose lipolysis to alleviate alcohol-induced steatosis in the liver. Also noteworthy is that alcohol cessation enhances hepatic fatty acid oxidation.

#### Hepatic autophagy

Alcohol cessation also resolves impaired hepatic autophagy, a key intracellular catabolic pathway that breaks down lipid droplets and other obsolete organelles. Chronic feeding of alcohol reduces the nuclear localization of transcription factor EB,^22^ which coordinates lysosome biogenesis with autophagy. Additionally, chronic alcohol feeding downregulates the activity of lysosomal acid lipase, causing intrahepatic lipid accumulation. Cessation of alcohol restores nuclear transcription factor EB levels to normal, thereby reactivating hepatic autophagy and the normal turnover of lipid droplets.^22^

#### Alcohol cessation and recovery following intragastric alcohol administration

Yin et al. (1988) examined recovery in rats subjected to intragastric alcohol feeding, during which rodents are given continual intragastric infusion of an alcohol diet through an inserted cannula.^26^ Liver damage in these animals is typically greater than in animals given oral feeding of alcohol ad lib. Alcohol removal for 2 weeks nearly normalized all liver functions in rats previously subjected to 6 weeks of intragastric alcohol administration.^26^

The foregoing findings indicate that several cellular mechanisms collectively contribute to resolution of steatosis and liver injury following alcohol cessation. First, since alcohol cessation would terminate ethanol metabolism, oxidant generation would be greatly decreased. Second, cessation normalizes circulating NEFA, their uptake by liver cells, and their reesterification into triglycerides. Third, alcohol cessation reactivates hepatic autophagy by restoring nuclear transcription factor EB levels, allowing resumption of lipid droplet degradation and organelle turnover. Interestingly, although alcohol cessation alleviates fat accumulation, it does not completely reverse fatty liver, probably because the amount of residual fat in livers of alcohol-fed rats overwhelms the degradation/oxidative systems. The latter findings indicate a longer recovery period is necessary to reverse fatty liver completely in alcohol-withdrawn rats.

## GI TRACT AND ALCOHOL

### Alcohol Metabolism in the GI Tract

As the principal site of alcohol absorption, the GI tract plays a particularly significant role in mediating the toxic effects of alcohol on the liver and other organs. GI metabolism of alcohol is significant as it affects the systemic availability of alcohol while it locally generates acetaldehyde. GI mucosal ADH catalyzes alcohol oxidation, especially in the oropharynx and esophagus where ADH class IV activity is relatively high, and it likely contributes to local toxicity because of the acetaldehyde it produces.^27^

Before alcohol reaches the liver, the stomach lining is the principal site of “first pass” metabolism of the ingested alcohol.^27^ Various isoforms of gastric ADH oxidize a significant percentage of ingested alcohol before it enters the portal circulation. The total first-pass metabolism of alcohol was calculated to be in the range of 7% to 9% and is influenced by many factors including gastric emptying.^28^ Besides ADH, the other major enzymes that catalyze alcohol oxidation, CYP2E1 and catalase, are present in GI mucosal cells. Similar to liver, GI CYP2E1 content also increases after chronic alcohol administration. GI tract microflora, including bacteria and yeast, possess ADH activity and metabolize alcohol to produce acetaldehyde, but they also are capable of generating alcohol during fermentation.^27^ Other factors such as motility, absorption, dilution by GI secretions, and rediffusion of alcohol all influence alcohol clearance from the GI tract. In addition, gender, age, genetics, and gastric morphology modulate gastric ADH activity. ADH levels are significantly lower in younger women compared with age-matched men. This difference probably accounts for greater alcohol-induced liver toxicity in women.^27^

### GI Injury and Recovery After Alcohol Exposure in Humans

Alcohol consumption interferes with the function of all parts of the GI tract. These malfunctions are due to the local production and systemic levels of acetaldehyde. Chronic alcohol use also damages and erodes the upper GI mucosa, which encounter undiluted alcoholic beverages, causing hemorrhagic lesions and increasing the risk of cancer development. Alcohol also impairs the muscles surrounding the stomach, small intestine, and large intestine. This affects motility, which, while delaying gastric emptying, shortens transit time in the small intestine, causing diarrhea. Essentially, alcohol inhibits absorption of a variety of nutrients by the small intestine and contributes to malnourishment commonly seen in patients with alcohol use disorder (AUD).^29^

#### Intestinal barrier disruption

Most relevant, chronic alcohol use disrupts the tightly regulated gut barrier function. This barrier consists of a system of highly specialized, intercellular, multiprotein junctional complexes known as tight junctions. These are located at the apical (luminal) ends of intestinal epithelial cells. Studies reveal that alcohol metabolism in the gut disrupts tight junction structural integrity. The consequent loss of the mucosal barrier allows paracellular translocation of pathogenic molecules—including cell wall components from gram-positive and gram-negative bacteria and fungi—into the general circulation, allowing direct access to the liver via the portal vein. Once inside the liver, these microbial components can activate resident macrophages (Kupffer cells) to initiate a necroinflammatory cascade. Alcohol compromises tight junction integrity by the following molecular mechanisms: generating reactive oxygen species, upregulating production of specific micro-RNAs, and disrupting both the epithelial cell methionine metabolic pathway and the intestinal circadian rhythm.^29^ In addition to the physical barrier, there are immunological and chemical barriers on the luminal surface of the GI tract. The chemical barriers secreted by the epithelial/immune cells include secretory immunoglobulin A, mucins, and antimicrobial peptides, all of which are altered by alcohol metabolism**.**

#### Alterations in the microbiota

A symbiotic balance between proinflammatory and commensal bacteria allows only trace amounts of luminal antigens to penetrate the intestinal barrier and enter the portal vein and systemic circulation. However, chronic alcohol administration alters the balance among intestinal microbiota. This is characterized by both quantitative and qualitative changes, including suppression of many commensal probiotic bacteria, vital for bile acid metabolism and for the generation of short- and long-chain fatty acids necessary for maintaining gut health and liver homeostasis.^30^

#### Recovery after abstinence

Recent studies have shown that a 3-week abstinence following the removal of alcohol induces a complete recovery of gut barrier function in subjects with AUD who presented with high intestinal permeability.^31^ Similar results were shown by other laboratories that reported a decrease in endotoxemia following the removal of alcohol.^16^ However, 3-week abstinence produces only an incomplete recovery of the gut microbiota,^31^ indicating that alcohol consumption has a more long-lasting effect on gut dysbiosis, even after more than 1 month of abstinence.^32^ A 3-week abstinence also increases bacterial populations known to be beneficial, which leads to a decrease in potential toxins and an increase in beneficial microbial metabolites.^31^

### GI Injury and Recovery After Alcohol Exposure in Animals

Most studies conducted to date using animal models have examined whether external agents—such as antibiotics, probiotics, prebiotics, synbiotics, betaine, zinc, indole-3-acetic acid, and long- and short-chain fatty acids—prevent or reverse alcohol-induced changes in the gut and prevent liver damage. Only one animal study has shown that sobering for 24 hours after 4 weeks of alcohol feeding partially restored intestinal barrier function, but such cessation did not reduce the inflammatory response in the colon.^33^

## PANCREAS

### Alcohol Metabolism in the Pancreas

Although the pancreas expresses both ADH and CYP2E1, its capacity for oxidative alcohol metabolism is significantly lower than that of the liver.^34^ However, the pancreas has a high capacity for nonoxidative alcohol metabolism, which is catalyzed by fatty acid ethyl ester (FAEE) synthases. These enzymes generate FAEE by condensing alcohol with a fatty acid (e.g., oleic acid). FAEE can bind to and accumulate in mitochondria to impair cell function in the pancreas and the heart,^35^ which is also rich in FAEE synthases.

### Pancreatic Injury and Repair After Chronic Alcohol Use in Humans

The association between alcohol consumption and pancreatic diseases has been recognized for more than 100 years. The pancreas contains two functionally distinct compartments: As an endocrine gland, the pancreas secretes insulin and glucagon, the hormones that govern glycemia. As an exocrine gland, the pancreas produces zymogen precursors of digestive enzymes used for food breakdown in the gut. Both compartments can suffer consequences of chronic alcohol use.

#### Pancreatitis

Chronic alcohol use is commonly associated with pancreatitis, a necroinflammatory disease of the exocrine pancreas that is classified as either acute or chronic. Although the association between chronic alcohol use and pancreatitis has long been recognized, the mechanism or mechanisms by which chronic alcohol use predisposes the pancreas to disease are not entirely understood. Despite this association, chronic alcohol use alone is not sufficient to trigger a clinical event, such as development of acute pancreatitis.^36^ Heavy drinking is believed to sensitize the pancreas to injury, whereas other factors trigger necroinflammation.

In developed countries, chronic alcohol use is the second most common factor associated with acute pancreatitis.^37^ In up to 20% of the cases, there are severe clinical complications of pancreatitis with mortalities of up to 10%.^37^

In contrast, in the Western world, chronic alcohol use is the major etiological factor in chronic pancreatitis, accounting for approximately 70% of reported cases. Alcohol-induced chronic pancreatitis is thought to have an early stage associated with recurrent attacks of alcohol-induced acute pancreatitis and a late stage characterized by steatorrhea, diabetes, fibrotic scarring, and pancreatic calcification. In many cases, it appears that alcohol-induced acute pancreatitis progresses to chronic pancreatitis. This progression is generally associated with frequent, severe, and acute attacks that are common among chronic alcohol users. Little is known regarding the effects of alcohol in humans after pancreatic damage. Because chronic pancreatitis is commonly associated with recurrent attacks of acute pancreatitis, it appears that continued alcohol consumption impairs proper pancreatic repair. In support of this, one study investigated pancreatic dysfunction associated with alcohol-induced chronic pancreatitis and demonstrated that pancreatic function deteriorated more slowly in patients who quit drinking compared with those who continued heavy drinking. These findings indicate that the functional deterioration of the pancreas associated with alcohol-induced chronic pancreatitis continues even after drinking ceases, although this occurs to a lesser degree than in those who continue to chronically use alcohol.^38^ A long-term, population-based study demonstrated that progression from acute to chronic pancreatitis is most common among chronic alcohol users. These findings indicate that alcohol consumption delays the normal repair process following acute pancreatitis and it may enhance the progression from acute to chronic pancreatitis. Although more work must be done to determine how alcohol affects repair of the pancreas, it appears that cessation of chronic alcohol use slows progression of alcohol-induced chronic pancreatitis.

### Pancreatic Injury and Repair After Alcohol Exposure in Animals

The structural and functional regeneration of the pancreas after acute injury is supported by studies of experimentally induced pancreatitis in rodents.^39^ One of the main characteristics of alcohol-induced chronic pancreatitis is the aberrant repair of injury that results in fibrotic scarring. Given the close association between chronic alcohol use and chronic pancreatitis, it is reasonable that chronic alcohol consumption adversely affects pancreatic repair. Using the Lieber-DeCarli pair-feeding model of alcohol administration in rats, one group reported that chronic alcohol feeding for 2 to 8 weeks significantly decreased the amylase content of the pancreas after cerulein-induced pancreatitis, indicating that alcohol consumption impaired functional pancreatic regeneration. This treatment did not affect total protein, DNA, or RNA content of the pancreas. Although no histological evaluation was performed, and amylase production declined, these authors concluded that alcohol consumption does not affect pancreatic regeneration.^40^ In contrast, Pap et al. reported that intragastric alcohol feeding for 2 months slowed the restoration of pancreatic weight and enzyme content in rats with surgically induced pancreatic injury.^41^ During this period, alcohol-fed animals developed chronic calcifying pancreatitis. Cessation of alcohol feeding resulted in structural and functional recovery of the pancreas. These results indicate that inhibition of pancreatic regeneration by alcohol is necessary to maintain the state of chronic pancreatitis. Cholecystokinin is a crucial peptide hormone in pancreatic regeneration. Alcohol feeding reduces cholecystokinin release and prevents pancreas regeneration after partial pancreatectomy.^42^ Additionally, using a model of virally induced pancreatitis, it was demonstrated that alcohol administration to mice delays pancreas repair.^43^ Together, these studies indicate that alcohol delays the structural repair and functional restitution of pancreatic tissue in animal models of alcoholic pancreatitis. Most studies indicate that cessation of alcohol consumption by rodents restores pancreatic structure and function.

## HEART

### Alcohol Metabolism in the Heart

Cardiac tissue expresses both major alcohol-metabolizing enzymes: ADH and CYP2E1.^44^ There are reports that both enzymes may influence alcohol-induced myocardial damage by converting alcohol to acetaldehyde. However, the heart also has a rich content of FAEE synthases, suggesting that nonoxidative alcohol metabolism prevails in this organ.

### Cardiac Injury and Recovery After Alcohol Exposure in Humans

Alcohol-induced dilated cardiomyopathy is an important manifestation of chronic alcohol use. Chronic AUD is accompanied by a high incidence of cardiac morbidity and mortality due to development of alcoholic cardiomyopathy. Cardiomyopathy can be seen by ventricle dilation, along with a reduced ventricular wall thickness and some contractile dysfunction. Alcohol/acetaldehyde toxicity along with mitochondrial production of reactive oxygen species is one theory proposed for alcohol-induced cardiac injury. Indeed, acetaldehyde can directly impair cardiac contractile function, disrupt cardiac excitation-contraction coupling, and promote oxidative damage and lipid peroxidation. Some resulting effects are oxidative injury, apoptosis, impaired myofilament Ca^2+^ sensitivity, impaired protein synthesis, and altered fatty acid extraction and deposition, along with changes in protein catabolism.^45^ The removal of alcohol is associated with the reduction or disappearance of myocardial damage and the improvement of function.^46^ A study on cardiovascular changes during different phases following the removal of alcohol found that heart rate, systolic blood pressure, and diastolic blood pressure were higher in the early stage of alcohol cessation. These cardiovascular parameters returned to baseline levels after 1 month of abstinence.^47^ Other cardiac effects of chronic alcohol exposure are cardiac arrhythmias (irregular heartbeat), tachycardia (fast heartbeat), and other cardiovascular disease. These cardiovascular parameters also returned to baseline levels after 1 month of abstinence.^47^ There is no evidence for reversal of cardiac fibrosis in humans with alcoholic cardiomyopathy. However, cessation of alcohol consumption can result in significant improvement in left ventricular function.^48^,^49^ In a case study, Mahmoud et al. showed that a patient who exhibited signs of alcoholic cardiomyopathy demonstrated severe global left ventricular systolic dysfunction with an ejection fraction of 20%.^50^ Moreover, the end-systolic dimension was 4.1 cm and the end-diastolic dimension was 5.0 cm. However, after 1 month of alcohol abstinence, this patient was asymptomatic, with a higher ejection fraction of 62%. The patient’s end-systolic dimension was 3.3 cm, and the end-diastolic dimension was 4.8 cm.^50^ Cardiac arrhythmias may explain cases of sudden death in patients with AUD who are abstinent.

The QTc interval (a measure of heart rate) is frequently prolonged during alcohol cessation syndrome and tends to become normal over time.^51^ The frequency and nature of arrhythmias, as well as some irregularities of their time-course due to alcohol cessation terms were studied in subjects with chronic AUD. Sinus tachycardia, abnormal excitation, and conduction were more frequently observed in the acute (early) period of alcohol cessation. In most cases, these symptoms ceased within 2 weeks after cessation.^52^

### Cardiac Injury and Recovery After Alcohol Exposure in Animals

Alcoholic cardiomyopathy is a specific heart muscle disease caused by chronic alcohol intake and has been studied in animal models. Chronic alcohol intake tends to increase left ventricular mass and dilatation that leads to heart failure in a rat model of alcohol administration. In one study, the authors postulated that alcohol intake activates the pro-renin receptor and contributes to cardiac remodeling and damage.^53^ They examined the relationship between the pro-renin receptor and alcoholic cardiomyopathy and found that alcohol intake increases myocardial fibrosis, myocardial oxidative stress, and inflammation response like that seen in humans. Studies examining recovery of cardiac function in animal models have not been described.

## BONE

### Alcohol Metabolism in Bone

It is not clear whether bone tissue itself metabolizes alcohol by oxidative metabolism (i.e., ADH and CYP2E1 catalysis) or by esterification with fatty acids. Current evidence supports that alcohol alone is the causative agent that delays bone growth and repair.

### Bone Injury and Repair After Alcohol Exposure in Humans

#### Osteopenia

Continued heavy alcohol use decreases bone density. The pathogenesis of osteopenia in AUD remains unclear, and many alcohol-related abnormalities have been proposed to explain bone loss.^54^ A direct inhibitory effect of alcohol on osteoblast function was suggested by in vivo and in vitro studies. The rapid increase in serum bone Gla protein (BGP) concentrations following alcohol cessation suggests that low serum BGP concentrations in heavy-alcohol users may result from a direct toxic effect of alcohol on osteoblast function and/or numbers.^54^ The role of alcohol as a risk factor for osteopenia was studied in subjects with AUD who did not have liver cirrhosis. The data show that chronic alcohol ingestion induces osteopenia regardless of whether liver cirrhosis is present, and that some relationship can be expected between the amount and duration of alcohol consumption and the degree of bone loss. Low serum levels of BGP in drinkers are reversible upon alcohol cessation, suggesting that reduction of osteoblast activity is likely the main factor responsible for alcohol-associated bone disease.^55^ Alcohol not only promotes bone loss but also impairs bone formation. Plasma concentrations of osteocalcin, a marker of bone formation, were measured in human male heavy drinkers before and after 3 weeks of alcohol cessation and compared with nondrinking men. Plasma osteocalcin levels in heavy-alcohol-using subjects were significantly lower than in controls. After 21 days of cessation, plasma osteocalcin levels were significantly higher than on the day of admission and were equal to those of controls, who did not have AUD. The results support the notion that the decrease of plasma osteocalcin with chronic alcohol use is reversible within 3 weeks following alcohol removal.^56^

#### Bone turnover

The biochemical markers for bone formation (osteocalcin, bone-specific alkaline phosphatase, and procollagen type 1 carboxy-terminal peptide) and resorption (c-terminal telopeptide and urine deoxypyridinoline) were studied in men who were heavy-alcohol users and in abstainers with more than 5 years of abstinence. The results were compared with male controls. The findings suggest that there is an imbalance between bone formation and bone resorption among heavy-alcohol users that results in rapid bone loss. Although most directions tended to normalize shortly following the removal of alcohol, biochemical data suggest that there may still be persistently high bone turnover after more than 5 years of abstinence.^57^

Although most studies suggest that alcohol induces bone loss, epidemiological studies indicate that higher bone mass is associated with moderate alcohol consumption in postmenopausal women. Therefore, a study investigated the hypothesis that moderate alcohol intake attenuates bone turnover after menopause. This study showed that abstinence from alcohol results in increased markers of bone turnover, whereas resumption of drinking reduces bone turnover markers. These results suggest that the inhibitory effect of alcohol on bone turnover attenuates the detrimental skeletal consequences of excessive bone turnover associated with menopause.^58^ Taken together, these studies indicate that alcohol has a direct effect on bone formation and resorption and that these effects are reduced during abstinence.

### Bone Injury and Repair After Alcohol Exposure in Animals

Animal (rodent) studies report that the adverse effects of alcohol on bones are limited not only to bone formation and resorption, but that chronic alcohol administration also impairs the healing capacity of fractured bone in rodents.^59^ In vitro studies report that proliferation of alcohol-exposed osteoblasts (precursor bone cells) is impaired and that such treatment enhances oxidant stress by increasing intracellular superoxide, which inhibits osteoblast proliferation.^60^,^61^ Recent in vivo studies suggest that oxidant stress inhibits bone repair, as fracture healing is restored in alcohol-fed rats treated with the antioxidant, *N*-acetylcysteine.^62^ Given the latter findings, it is reasonable to postulate that alcohol cessation may fully restore osteogenesis in bone.

## SUMMARY

Continual heavy alcohol consumption damages multiple organs/systems. This review focused on damage and recovery in five of those tissues in humans and experimental rodents. The greatest degree of alcohol-induced injury occurs in the liver and GI tract, as both these organs/systems are the first to encounter high concentrations of imbibed alcohol. The liver and GI tract are well equipped to oxidatively metabolize alcohol. However, alcohol oxidation comes at a cost, as it generates acetaldehyde, which is capable of forming toxic acetaldehyde-macromolecular adducts as well as free radicals that oxidize lipids and form reactive lipid peroxides. Thus, the continual metabolic generation of these intermediates eventually disrupts homeostasis, causing cell death, inflammation, and the eventual breakdown of organ integrity.

In the pancreas and heart, alcohol is minimally oxidized. Instead, most of it is esterified with fatty acids, forming FAEE. These molecules bind to mitochondria and disrupt the generation of energy that is normally reserved for pancreatic secretion or myocardial contraction. Although it is not clear whether oxidative or nonoxidative alcohol metabolism actually occurs in bone tissue, it is clear that alcohol exposure to osteoblasts inhibits their proliferation by causing oxidant stress. Also, structural weakening of bone and delays in fracture healing are clearly evident after chronic alcohol consumption by rodents.

**Figure 1:**
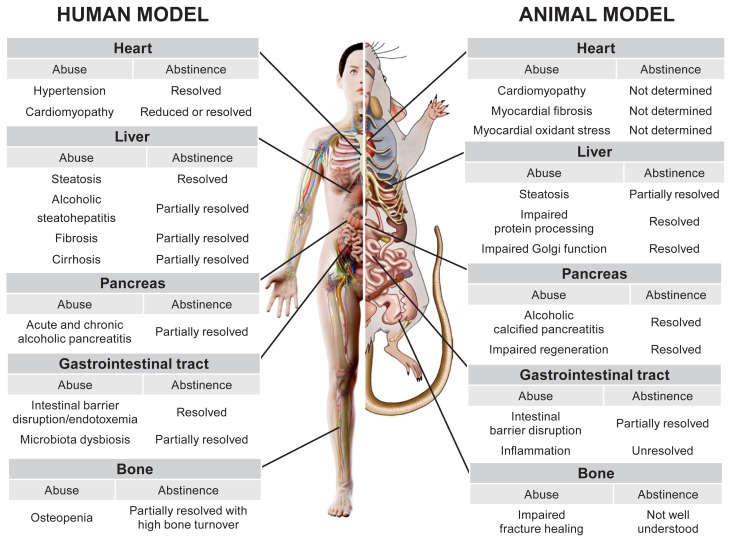
Schematic diagram of the effects of chronic alcohol use and abstinence in humans and rodents on various organs and systems, including the heart, liver, gastrointestinal tract, pancreas, and bone. Each row describes a consequence of chronic alcohol use, whether it is resolved by abstinence, and, if so, to what degree. Adapted with permission from SciePro/stock.adobe.com (human) and Science Photo Library, London (rodent).

Despite alcohol-induced damage to these tissues, abstinence, in its simplest form, brings about either complete or partial recovery, but the extent of such recovery depends on the extent of the damage, as shown in Figure 1. For example, it is unlikely that abstinence would be effective in a case of decompensated cirrhosis, but resolution of cirrhosis which involves a portion of the liver (i.e., compensated cirrhosis) is more likely. Thus, the examples provided in this review highlight the value of intrinsic regenerative processes that maintain organ function.

Finally, more basic research is needed to clearly evaluate whether abstinence that follows chronic alcohol consumption completely or partially restores the full integrity of the affected organs. To date, the results appear promising that cessation of alcohol consumption indeed allows partial or full recovery, depending on the parameter being measured. It is also worth noting that alcohol-induced pathology in animals (usually rodents) does not fully reflect the extent of injury incurred by human heavy drinkers. However, the use of other feeding models, such as intragastric feeding and the acute-on-chronic feeding model have yielded valuable information on liver damage in animals that consume similar amounts of alcohol and have similar drinking patterns as humans with AUD.

## FINAL REMARKS

The focus of this review has been on organ recovery after cessation of chronic alcohol use. Abstaining from alcohol by a person with AUD is not a trivial matter. A recent review by Asrani et al. gives important details on the scope of the global burden of alcohol-associated disease;^63^ although its principal focus is ALD, it applies to all the alcohol-induced disorders described here. Presently, the problems of alcohol-related morbidity (suffering from AUD) and mortality (death from AUD) are rising worldwide. Their reductions will require multifaceted solutions that focus on early identification of problem drinking and interventions at the population level (e.g., increased taxation of beverages; youth education) and at the patient level (e.g., early diagnosis of organ injury; counseling by an addiction specialist). Although none of the aforementioned examples, by themselves, are considered innovative, their combined use represents a new approach, especially when they make use of technological advances, including smartphone technology and telehealth. The team approach to treatment is important because, although a physician can diagnose and treat organ injury, an addiction specialist or mental health professional also must be part of the treatment plan to prevent patient relapse. These measures, along with public reeducation about social stigmas related to alcohol addiction, will likely reverse the rising trends toward heavy drinking.
